# CD81 extracted in SMALP nanodiscs comprises two distinct protein populations within a lipid environment enriched with negatively charged headgroups

**DOI:** 10.1016/j.bbamem.2020.183419

**Published:** 2020-11-01

**Authors:** Hoor Ayub, Michelle Clare, Ivana Milic, Nikola P. Chmel, Heike Böning, Andrew Devitt, Thomas Krey, Roslyn M. Bill, Alice J. Rothnie

**Affiliations:** a College of Health & Life Sciences, Aston University, Aston Triangle, Birmingham B4 7ET, UK; b Department of Chemistry, University of Warwick, Gibbet Hill Road, Coventry CV4 7AL, UK; c German Center for Infection Research (DZIF) Hannover-Braunschweig Site and Institute of Virology, Hannover Medical School, 30625 Hannover, Germany; d Center of Structural and Cell Biology in Medicine, Institute of Biochemistry, University of Luebeck, Luebeck, Germany; e Centre for Structural Systems Biology (CSSB), Hamburg, Germany

**Keywords:** CD, circular dichroism, DLS, dynamic light scattering, DDM, dodecylmaltoside, EC2, extracellular loop 2, ELISA, enzyme linked immune absorbant assay, EM, electron microscopy, HCV, hepatitis C virus, HRP, horseradish peroxidase, LEL, large extracellular loop, mAb, monoclonal antibody, MS, mass spectrometry, Ni-NTA, nickel nitrilotriacetic acid, OG, octyl glucoside, OPD, *o*-phenylenediamine dihydrochloride, PBS, phosphate-buffered saline, PBST, phosphate-buffered saline + Tween 20 (0.05% v/v), *P. pastoris*, *Pichia pastoris*, SEL, small extracellular loop, SD, Standard deviation, SDS, sodium dodecyl sulphate, SDS-PAGE, SDS polyacrylamide gel electrophoresis, SEC, size exclusion chromatography, SEM, standard error of the mean, SMA, styrene maleic acid *co*-polymer, SMALP, styrene maleic acid lipid particle, Tetraspanin, SMALP, Membrane protein, Solubilisation, Purification, Stability

## Abstract

Tetraspanins exert a wide range of cellular functions of broad medical importance. Despite this, their biophysical characteristics are incompletely understood. Only two high-resolution structures of full-length tetraspanins have been solved. One is that of human CD81, which is involved in the infectivity of human pathogens including influenza, HIV, the malarial Plasmodium parasite and hepatitis C virus (HCV). The CD81 crystal structure identifies a cholesterol-binding pocket, which has been suggested to be important in the regulation of tetraspanin function. Here we investigate the use of styrene-maleic anhydride co-polymers (SMA) for the solubilisation and purification of CD81 within a lipid environment. When CD81 was expressed in the yeast *Pichia pastoris*, it could be solubilised and purified using SMA2000. This SMALP-encapsulated CD81 retained its native folded structure, as determined by the binding of two conformation-sensitive anti-CD81 antibodies. Analysis by size exclusion chromatography revealed two distinct populations of CD81, only one of which bound the HCV glycoprotein, E2. Optimization of expression and buffer conditions increased the proportion of E2-binding competent CD81 protein. Mass spectrometry analysis indicated that the lipid environment surrounding CD81 is enriched with negatively charged lipids. These results establish a platform to study the influence of protein-lipid interactions in tetraspanin biology.

## Highlights

• CD81 expressed in *Pichia pastoris* can be solubilised and purified using SMA polymer.
• SMALP-encapsulated CD81 retains native folded structure.
• Expression and buffer conditions can be optimized to improve protein quality.
• The lipid environment surrounding CD81 is enriched with negatively charged lipids.

## Graphical abstract

**Figure:**
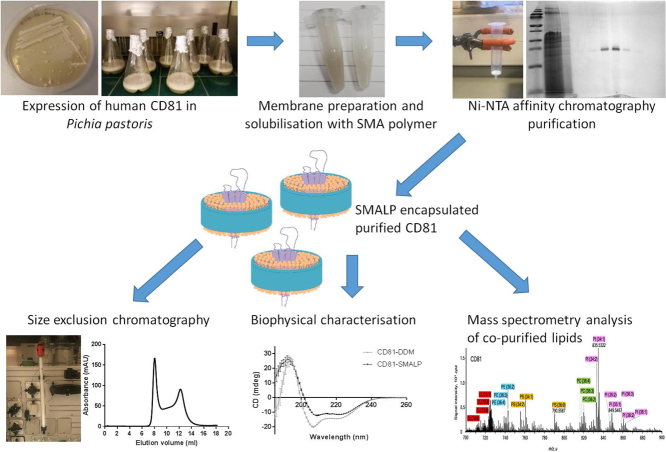

## 1 Introduction

CD81 is a member of the tetraspanin family of membrane proteins that have broad biological and medical importance. Tetraspanins engage in a wide range of incompletely-understood molecular interactions, exerting biological functions in cell-cell adhesion, cell proliferation, the immune system and during fertilization. They coordinate the trafficking of molecules into tetraspanin-enriched membrane microdomains thereby affecting cell signalling, morphology, motility and fusion [1]. Several tetraspanins have well-documented roles in infectious disease processes [2], while the expression of others correlates with tumour stage, type and patient outcome by affecting cell growth, morphology, invasion and metastasis [3].

There are 34 tetraspanin family members in mammals, of which 33 have been identified in humans [1]. Tetraspanin proteins have four transmembrane domains (TM1–4), intracellular N- and C-termini and two extracellular domains, one small (known as EC1 or SEL) and one large (typically 100 residues; known as EC2 or LEL). The family's signature motif is the presence of four or more cysteine residues in LEL; two in highly-conserved ‘CCG’ motifs [1]. Central to tetraspanin function are their extensive associations with an array of signalling proteins [1]; tetraspanin homodimers have been proposed to be the building blocks for the assembly of multicomponent tetraspanin protein complexes [4,5]. The 6 Å cryo-electron microscopy structure of a uroplakin tetraspanin revealed a rod-shaped structural morphology consisting of four TM helical bundles bound to a single TM helix partner [6].

Only two high-resolution structures are available of full-length tetraspanins: human CD81 [7] and human CD9 [8]. The 2.95 Å crystal structure of CD81 confirms the presence of the conserved CCG motif and two disulfide bridges [7]. CD81 is involved in cell-cell adhesion, cell proliferation, the immune response, fertilization and the infectivity of several important human pathogens including influenza, HIV, the malarial Plasmodium parasite and hepatitis C virus (HCV) [9]. It forms diverse, associations with other proteins in cell membranes: the oligomeric status of CD81 within these complexes and the mechanistic detail of how they exert their biological function remain incompletely understood. The CD81 crystal structure identifies a cholesterol-binding pocket; cholesterol binding appears to modulate CD81 activity in cells and has been suggested as a potential mechanism for the regulation of tetraspanin function [7] prompting a need to understand the role of the membrane lipid environment in CD81 folding and function.

Biological membranes are complex mixtures of membrane proteins and lipids. To study the function of an individual membrane protein often requires purification of that protein, however purification of membrane proteins can be challenging. This is largely due to their location within the lipid bilayer. First the protein needs to be extracted from the bilayer, and then the hydrophobic regions of the protein, which would normally interact with lipid acyl chains, need to be stabilised in some way to allow the protein to be soluble in water and not just aggregate. Typically this has been achieved with the use of mild detergents, such as octyl glucoside (OG) or dodecylmaltoside (DDM). These detergents disrupt the membrane bilayer and then form a micelle around the hydrophobic parts of the protein. Detergents have proven successful for the study of many proteins, including their use in solving the X-ray crystal structure of CD81 [7]. However one of the downsides of detergents is that during solubilisation they strip away the native lipid environment of a membrane protein. It is well established that specific lipid environments are important not just for stability of many proteins but also for their function [[10], [11], [12]], including CD81. In recent years an alternative approach to detergents has been developed using styrene maleic acid *co*-polymer (SMA), which extracts membrane proteins with their surrounding lipids [[13], [14], [15]]. The SMA polymer inserts into a membrane and extracts a disc of lipid bilayer with the polymer wrapping around the outer edge [16]. This nanodisc-like structure is termed an SMA lipid particle (SMALP), and the approach allows for the extraction and purification of membrane proteins without ever removing them from their natural lipid environment [13,[17], [18], [19]]. Given the proposed importance of lipids for the function of CD81, in this study we aimed to investigate the use of SMA polymers for the solubilisation and purification of the tetraspanin CD81.

## 2 Materials & methods

### 2.1 Materials

*Pichia pastoris* wild type strain X-33 (Invitrogen) was electroporated to integrate the hCD81 expression plasmid comprising pPICZB encoding C-terminus His_6_ tagged CD81 [20]. SMA 2000 co-polymer (2:1 Cray Valley) and SZ25010 co-polymer (3:1 Polyscope) were obtained as styrene maleic anhydride and hydrolysed to styrene maleic acid forms as described previously [21]. Detergent n-dodecyl-B-D-maltoside (DDM), biotin, zeocin, yeast extract, peptone, yeast nitrogen base and agar antibiotic were purchased from ThermoFisher Scientific. Ni^2+^-NTA resin for IMAC purification was purchased from Qiagen and Bio-Rad Econo-column empty chromatography column was used. Mini EDTA-free Protease Inhibitor Cocktail Tablets from Roche Applied Science. Anti-mouse HRP-conjugated secondary antibody was purchased from Cell Signalling Technology. Ammonium formate was purchased from Sigma Aldrich. Methanol, chloroform and ultra pure water, all LC-MS grade, were purchased from Fisher, UK. Anti-CD81 antibodies 2s131, 1s337 and 1s135 were generated as described previously [22] and hybridoma supernatants were used for all ELISA experiments.

### 2.2 Expression of CD81

*P. pastoris* transformed with CD81 was streaked onto YPD-Zeocin agar plates (1% (w/v) yeast extract, 2% (w/v) peptone, 2% (w/v) glucose, 2% (w/v) agar, 100 mg/ml Zeocin) from a −80 °C glycerol stock. The plates were incubated at 30 °C for three days or until single colonies were visible.

For large scale recombinant CD81 expression, yeast growth was performed using baffled shake flasks. One colony of *P. pastoris* from YPD agar plates was added to a 250 ml baffled flask containing 50 ml BMGY (1% (w/v) yeast extract; 2% (w/v) peptone, 100 mM potassium phosphate pH 6.0, 1.34% (w/v) YNB, 4 × 10^−5^% (w/v) biotin, 1% (v/v) glycerol) and incubated overnight at 30 °C and 220 rpm. 5 ml of this seed culture were added into 200 ml of fresh BMGY media in separate 1 l baffled flask and grown for 22–24 h at 30 °C and 220 rpm. To induce recombinant CD81 expression, cell density was monitored until an OD_600_ of 1 was reached. Cells were then pelleted by centrifugation at 5000*g* for 10 min. The cells were resuspended in 500 ml BMMY media (1% (w/v) yeast extract; 2% (w/v) peptone, 100 mM potassium phosphate pH 6.0, 1.34% (w/v) YNB, 4 × 10^−5^% (w/v) biotin, 0.5% (v/v) methanol) in a 2 l baffled shake flask. The culture was grown for 22 h, supplemented with 5 ml of absolute methanol and then grown for a further 22 h. Cells were harvested by centrifugation (5000*g* for 20 min at 4 °C) and stored at −80 °C until further use.

### 2.3 Membrane preparation

The cells from large scale *P. pastoris* growth were lysed using an Emulsiflex-C3 cell disrupter (Avestin) to isolate membranes. The cell pellet was mixed with ice-cold breaking buffer (5% (v/v) glycerol, 2 mM EDTA, 100 mM NaCl, 50 mM NaH_2_PO_4_, 50 mM Na_2_HPO_4_, pH 7.4) supplemented with protease inhibitors, at a ratio of 3 ml buffer per gram of cell pellet. The cells were passed through the cell lyser 5 times (for ≈15 min) at 20,000 to 25,000 psi. Any unbroken cells and cell debris were removed by centrifugation (10,000*g*, for 30 min at 4 °C). The supernatant was collected and ultracentrifuged (100,000*g*, for 1 h at 4 °C). The pellet containing yeast membranes was collected, resuspended in buffer A (20 mM HEPES, 50 mM sodium chloride, 10% (v/v) glycerol, pH 7.0) at a concentration of 160 mg/ml (wet pellet weight) and homogenised using a glass homogeniser. Membrane fractions were stored at 4 °C for immediate use or at −80 °C for future analysis.

### 2.4 Solubilisation & purification

For initial solubilisation trials, CD81-containing *P. pastoris* membranes, at 160 mg/ml (wet pellet weight) were diluted four fold with Tris-HCl purification buffer (20 mM Tris-HCl, 150 mM sodium chloride, pH 8.0), and incubated with varying concentrations of SMA 2000 polymer (0–5% w/v), SZ25010 polymer (2.5% w/v) or DDM (2% w/v), for time points ranging from 15 min to 4 h at room temperature, with gentle shaking. Turbidity was monitored by measuring the OD_600_ of the sample. Insoluble material was sedimented by ultracentrifugation (100,000*g*, 20 min at 4 °C) to yield supernatant containing solubilised CD81. Samples of the solubilised protein within the supernatant and insoluble protein within the pellet were analysed by Western blotting, using an anti-CD81 primary antibody (2s131) at a dilution of 1:200, followed by an anti-mouse HRP-conjugated secondary antibody. Importantly no reducing agents were used in the sample buffer as this has been shown to prevent binding of the antibody [22].

For downstream purification, CD81-containing *P. pastoris* membranes, at 160 mg/ml (wet pellet weight) were diluted four fold with either Tris-HCl purification buffer (20 mM Tris-HCl, 150 mM sodium chloride, pH 8.0) or HEPES purification buffer (20 mM HEPES, 200 mM sodium chloride, 10% glycerol, pH 8.0) and incubated with 2.5% (w/v) of the SMA polymer for 1 h at room temperature with mild agitation, before harvesting the solubilised protein by ultracentrifugation (100,000*g*, 20 min at 4 °C).

The solubilised protein was incubated with Ni-NTA agarose resin overnight at 4 °C with mild agitation. Initial trials utilized 100 μl resin per ml of solubilised protein, while optimized conditions increased the volume of resin 5-fold. The solution was poured into an empty column and the flow-through was collected. Resin was washed with 50 bed volumes of purification buffer containing 20 mM imidazole, a second wash with 20 bed volumes of purification buffer containing 40 mM imidazole and a final wash with 1 bed volume of purification buffer containing 60 mM imidazole. The bound CD81 was eluted with 3 bed volumes of purification buffer containing 300 mM imidazole. The elution fractions were pooled and concentrated using a centrifugal concentrator (Vivaspin 20 kDa cut-off, Sartorius).

Alternatively CD81 membranes were solubilised with 1% (w/v) DDM and 0.1% cholesterol hemisuccinate in 250 mM NaCl, 20 mM HEPES pH 7.4, 20 mM imidazole, supplemented with 10% (v/v) glycerol, at 4 °C for 2 h before ultracentrifugation (100,000*g*, 60 min at 4 °C). DDM solubilised protein was mixed with Ni-NTA resin (at a ratio of 1:20) for 2 h at 4 °C. The solution was poured into a chromatography column and the flow-through was collected. Resin was washed with 20 column volumes of buffer containing 100 mM NaCl, 20 mM HEPES pH 7.4, 1% glycerol, 0.1% (w/v) DDM, 0.01% CHS and 40 mM imidazole. The bound CD81 was eluted with 5 column volumes using the same buffer but with 300 mM imidazole. The elution fractions were pooled and concentrated using a centrifugal concentrator (Vivaspin 50 kDa cut-off, Sartorius).

Purified protein was quantified using an SDS-PAGE based assay with bovine serum albumin as a standard [23].

Purified CD81-SMALP was further analysed and purified through size exclusion chromatography (SEC) using an AKTA Pure system (GE Healthcare) and a Superdex increase 200 10/300 GL SEC column.

### 2.5 Expression & purification of HCV E2 glycoprotein

A full-length streptavidin-tagged HCV E2 ectodomain (sE2) of the clinical isolate UKN2b_2.8 was expressed in *Drosophila* S2 cells as previously described [24,25]. Briefly, *Drosophila* S2 cells were transfected, amplified, and induced with 4 μM CdCl_2_ at a density of ~8 × 10^6^ cells/ml for 6–9 days for large-scale production. Proteins were purified from the supernatant by affinity chromatography using a Strep-Tactin Superflow column (IBA, Goettingen, Germany) followed by SEC using a Superdex200 column (GE Healthcare, Uppsala, Sweden). Pure monomeric protein was concentrated to ~15 mg/ml for use in ELISA.

### 2.6 ELISA assays

For anti-CD81 binding ELISA, 50 μl purified protein (100 μg/ml) were added to a 96-well plate (Immulon II ELISA plate, Bunc) in triplicate and incubated overnight at 4 °C. Unbound protein was removed by washing three times with PBS (200 μl/well). Plates were blocked with 100 μl of 2% (w/v) bovine serum albumin in PBS per well for 20 min at room temperature to reduce any nonspecific binding and washed three times with PBS. Samples were incubated with 50 μl primary anti-CD81 antibodies 1s337, 1s135 or 2s337 [22], diluted 1:2 in PBS-Tween (0.05% v/v). After three PBS washes (200 μl/well), samples were incubated with HRP-conjugated anti-mouse secondary antibody (Sigma-Aldrich) at a dilution 1: 1000 in PBS-Tween for 1 h at room temperature. Three final washes were performed (200 μl/well) followed by the addition of SIGMA*FAST*™ OPD tablets in solution (Sigma-Aldrich) (100 μl/well) and incubated for 15 to 20 min at room temperature. The stop reagent (1 M sulphuric acid from Sigma-Aldrich – 100 μl/well) was used, once the solution started changing colour from colourless to pale orange. The absorbance was measured on a Fusion plate reader (Perkin-Elmer) at 492 nm.

An ELISA was also used to measure binding of HCV E2 glycoprotein to CD81. CD81 protein samples were bound to plates, blocked and washed as described above. Following this samples were incubated with 50 μl of 85 μg/ml purified streptavidin-tagged E2 protein for 1 h at room temperature before three PBS washes. 50 μl primary anti- Strep antibody (Progen #910STR) diluted 1:5,000 in PBST and incubated at room temperature for 1 h with gentle shaking. After three PBS washes (200 μl/well), samples were incubated with HRP-conjugated anti-mouse secondary antibody (Sigma-Aldrich) at a dilution 1: 1000 in PBST. Wells were washed and developed as described above.

### 2.7 Dynamic light scattering (DLS)

50 μl CD81-SMALP (0.05 mg/ml) or CD81-DDM (0.09 mg/ml) were used for the DLS measurement. The data were collected using a Malvern Instruments ZetasizerNano S (633 nm). Measurements were taken in disposable ultra-micro UV cuvettes (BrandTech Scientific) at 20 °C with 300 s equilibration time, where automated parameters were used. Each measurement was repeated seven times.

### 2.8 Circular dichroism spectroscopy (CD)

For circular dichroism (CD) analysis, the purified protein was buffer exchanged into 20 mM sodium phosphate buffer for CD81-SMALP. Sodium phosphate buffer was supplemented with 0.1% DDM and 0.01% cholesterol hemisuccinate for CD81-DDM purified protein. This was because high background signal was observed with the HEPES/NaCl buffer whereas minimum background noise was observed with sodium phosphate buffer. Data were collected between 260 and 180 nm (far-UV wavelength region) at 0.2 nm intervals at 20 °C using a Jasco J-1500 CD spectrometer. A 1 mm path length quartz cuvette (Starna UK) containing 200 μl CD81-SMALP (0.05 mg/ml) or CD81-DDM (0.09 mg/ml) was used for each analysis and 18 technical replicates were performed for each sample. For thermal melt analysis, temperature was increased from 25 to 90 °C, in increments of 5 °C. Lipid only SMALPs, using 1,2-dimyristoyl-*sn*-glycero-3-phosphocholine lipid, were used for background signal correction to subtract possible SMA co-polymer and lipid noise from the CD spectrum. The amount required as a control for CD analysis was calculated using the absorbance peak at 260 nm.

Structural CD data analysis was performed using Dichroweb [26,27] with the CDSSTR, Contin-LL and Selcon3 algorithms [[28], [29], [30], [31], [32], [33], [34]].

### 2.9 Protein aggregation assay

The aggregation of purified protein following heating at a range of temperatures from 20 °C to 90 °C was assayed. 100 μl of purified protein samples (30 μg/ml) were heated for 10 min at selected temperature points followed by centrifugation at 10,000*g* for 10 min to remove aggregation. Supernatant was collected and 20 μl sample from each were analysed by Western blotting, using an anti-CD81 primary antibody (2s131) at a dilution of 1:200, followed by an anti-mouse HRP-conjugated secondary antibody.

### 2.10 Negative stain electron microscopy (EM)

EM grids (copper grid with a formvar-carbon support film from Agar Scientific) were glow discharged for 2 min to distribute negative charge on the surface before sample adsorption. 10 μl purified protein (2 μg/ml) was placed on each grid and incubated for 2 min at room temperature. Excess solution was blotted off and grids were washed three times with distilled water before gently drying using Whatman filter paper (Agar Scientific). A 3 μl drop of 2% (w/v) uranyl acetate stain was placed on each grid and incubated for 4 min followed by three washes with distilled water. The grids were visualised using a JEOL 2100Plus super high resolution TEM-STEM at room temperature and images were acquired at 60,000×.

### 2.11 Lipid extraction

Lipids were extracted following a previously described method using methyl-*tert*-butyl ether [35] with slight modifications. Briefly, 50 μl sample was mixed with 375 μl methanol in a 2 ml polypropylene tube (Eppendorf, UK) and vortexed for 10 s. Methyl-*tert*-butyl ether (1,250 μl) was added and the sample was vortexed for an additional 10 s. Lipids were extracted for 1 h at 4 °C on a rotary shaker. Phase separation was induced by addition of 375 μl water (MS grade, Fisher, UK) followed by 10 s vortexing and 10 min extraction on a rotary shaker at 4 °C. Samples were centrifuged at 1000 ×*g* for 10 min, the upper phase was collected and dried under vacuum (vacuum concentrator, Eppendorf, Germany) and samples were stored at −20 °C prior to MS analysis.

### 2.12 Mass spectrometry

Lipid extracts corresponding to 150 μl of sample were dissolved in an electrospray (ESI) solution containing 5 mM ammonium formate in a mixture of methanol and chloroform (2:1, v:v) and analysed by mass spectrometry (MS). Each sample was directly infused into the 5600 TripleTof mass spectrometer (AB Sciex, UK) at a flow rate of 500 nl/min using a syringe pump (Harvard Appartaus, USA) and PicoTip ESI emitters (New Objective, Germany). Data were acquired in both positive (2.4 kV) and negative ion modes (−2.2 kV). MS and MS/MS spectra were acquired in an information dependent mode (IDA) for 15 min. One IDA cycle consisted of a MS survey scan (*m*/*z* range from 400 to 1250 positive ion mode, and *m*/*z* 200 to 1250 in negative ion mode) followed by consecutive CID fragmentations (10 V collision energy) of the six most abundant ions in MS survey scan. Acquired ions were temporarily excluded from MS/MS acquisition for 300 s.

### 2.13 Data analysis

Western blots and SDS-PAGE gels were analysed by densitometry using the software ImageJ.

Mass spectrometry data visualization and analysis was done using Peak View software (version 2.2, AB Sciex, UK). The ion list with precursor *m*/*z* values averaged over the acquisition time was exported to Lipid Maps®. All positive identifications were manually inspected for the presence of lipid-specific fragment ions or neutral losses.

## 3 Results

### 3.1 Solubilisation of CD81

**Fig. 1:**
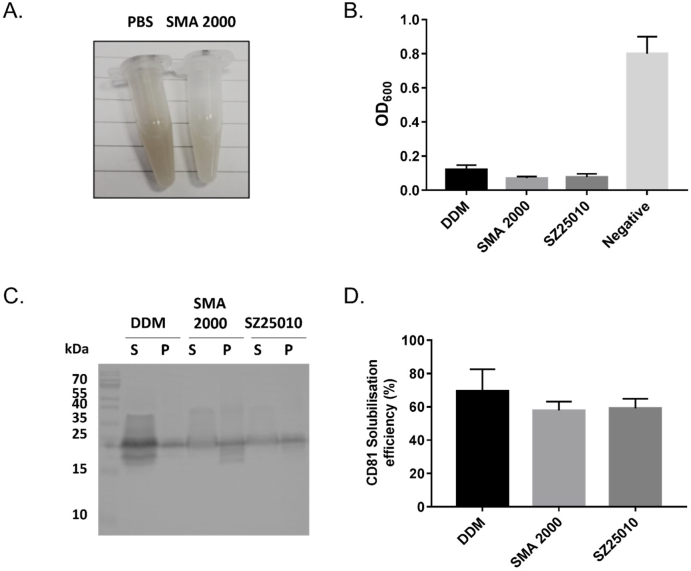
CD81 can be solubilised using SMA polymers or conventional detergent. Membrane preparations from CD81-expressing *P. pastoris* cells (30 mg/ml wet pellet weight) were solubilised with 2% (w/v) DDM, 2.5% (w/v) SMA polymer or PBS (negative control) for 1 h at room temperature. A) Image showing the change in appearance as SMA clarifies the membrane suspension compared to PBS control. B) Optical density measurements of the samples measured at 600 nm. Data are mean ± SD, n = 3. C) Following solubilisation, samples were centrifuged at 100,000*g* for 20 min at 4 °C. Solubilised protein in the supernatant (S) was harvested. Insoluble material in the pellet (P) was resuspended in an equal volume of purification buffer supplemented with 2% (w/v) SDS. Samples were analysed by Western blot using an anti-CD81 primary antibody (mAb 2s131). D) Average CD81 solubilisation efficiency. Data are mean ± SD, n = 3.

Recombinant CD81 was expressed in *P.* *pastoris* as described previously [20]. Membranes were extracted from these cells and the solubilisation of these membranes by two different SMA polymers, SMA 2000 or SZ25010 at 2.5% (w/v), was investigated and compared to the conventional detergent DDM (1% (w/v)). SMA 2000 and SZ25010 are both *co*-polymers of styrene and maleic acid but they differ in their ratio of styrene:maleic acid and average molecular weight. SMA 2000 has a 2:1 ratio of styrene:maleic acid with an average molecular weight of 7.5 kDa, whereas SZ25010 has a 3:1 ratio, and average molecular weight of 10 kDa. Both have been shown previously to be effective at solubilising membrane proteins [36]. As shown in Fig. 1A & B, the addition of either SMA polymer or DDM resulted in a dramatic drop in turbidity within 1 h, whereas no change in turbidity was observed with the negative control (PBS). To investigate if this general breakup of the membrane correlated with solubilisation of CD81 specifically, the sample was subjected to ultracentrifugation, and the solubilised protein in the supernatant (S) and insoluble protein in the pellet (P) collected and analysed by Western blot. In Fig. 1C it can be seen that DDM solubilisation yields a relatively intense band for CD81 in the soluble fraction. However, there is still some CD81 which remains insoluble, and overall DDM gives a solubilisation efficiency of approximately 70%. For both SMA 2000 and SZ25010 the band in the soluble fraction appears less intense than for DDM, and somewhat smeared. This finding supports previous suggestions that the presence of excess free SMA polymer interferes with binding to the antibody. However if the insoluble pellet fractions are examined (which do not contain the excess SMA polymer) they are of similar intensity to the pellet fractions obtained with DDM, suggesting that a substantial amount of the CD81 has been solubilised. In fact when the pellet fractions are compared to those obtained with a buffer negative control, it is apparent that both SMA 2000 and SZ25010 solubilised approximately 60% of the total CD81 (Fig. 1D). Therefore going forward the amount of CD81 remaining insoluble in the pellet was always measured and compared to the negative PBS control.

**Fig. 2:**
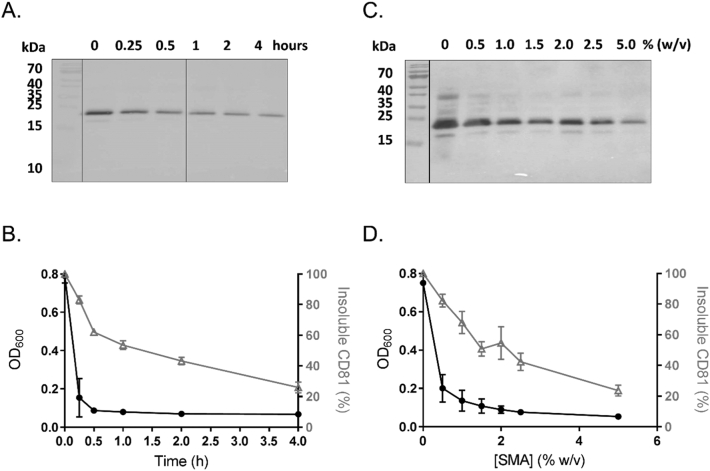
Solubilisation of CD81 by SMA 2000 is slower than the break-up of the total membrane. A) Representative Western blot showing the amount of CD81 remaining insoluble (in the pellet) after various lengths of incubation with 2.5% (w/v) SMA 2000 at room temperature. B) Average measurements of solubilisation at different time points. Optical density measurements at 600 nm (solid black circles). Percentage insoluble CD81 determined by densitometric analysis of Western blots as shown in A (open grey triangles). Data are mean ± SD, n = 3. C) Representative Western blot showing the amount of CD81 remaining insoluble after incubation with varying concentrations of SMA 2000 for 1 h at room temperature. D) Average measurements of solubilisation using different SMA 2000 concentrations. Optical density measurements at 600 nm (solid black circles). Percentage insoluble CD81 determined by densitometric analysis of Western blots as shown in C (open grey triangles). Data are mean ± SD, n = 2.

Having established that SMA polymers were capable of effectively extracting CD81 from *P. pastoris* membranes, the next step was to optimise the conditions for this extraction. As no difference was observed in the abilities of SMA 2000 and SZ25010 to extract CD81 (Fig. 1), and previous reports with other proteins suggested SZ25010 gives lower stability and yield in downstream purification, it was decided to proceed solely with SMA 2000, which had previously been shown to be the best polymer to date [36]. The first variable investigated was solubilisation time from 15 min to 4 h. As shown in Fig. 2B the membrane turbidity dropped rapidly and after just 15 mins remained constant. However, although this bulk membrane disruption occurred quickly, the specific solubilisation of CD81 was much slower (Fig. 2A & B), reaching its highest levels after 4 h. Secondly the concentration of SMA 2000 for optimal solubilisation was investigated, from 0.5% (w/v) to 5% (w/v). Fig. 2D shows that the optical density decreased substantially with just 0.5% (w/v) SMA 2000, and increasing the concentration made only small improvements to this. However, this was not replicated in CD81 specific solubilisation (Fig. 2C), which was only approximately 20% efficient with 0.5% (w/v) SMA 2000, and gradually increased as the concentration of SMA was increased. Thus it is clear that the changes in turbidity do not reflect the solubilisation of CD81. CD81 solubilisation by SMA 2000 was greatest after 4 h at room temperature, and the efficiency could be improved by increasing the concentration of SMA 2000 to 5% (w/v) SMA. Although these results show it may be possible to increase the yield of CD81-SMALPs by increasing the concentration of SMA or solubilisation time, the effects of excess SMA [37,38], or keeping CD81 at room temperature for longer periods would need to be evaluated. For these reasons, it was decided going forward to use standard conditions of 2.5% (w/v) SMA 2000 for 1 h at room temperature, which achieved a solubilisation efficiency of approximately 60%.

### 3.2 Purification of CD81

**Fig. 3:**
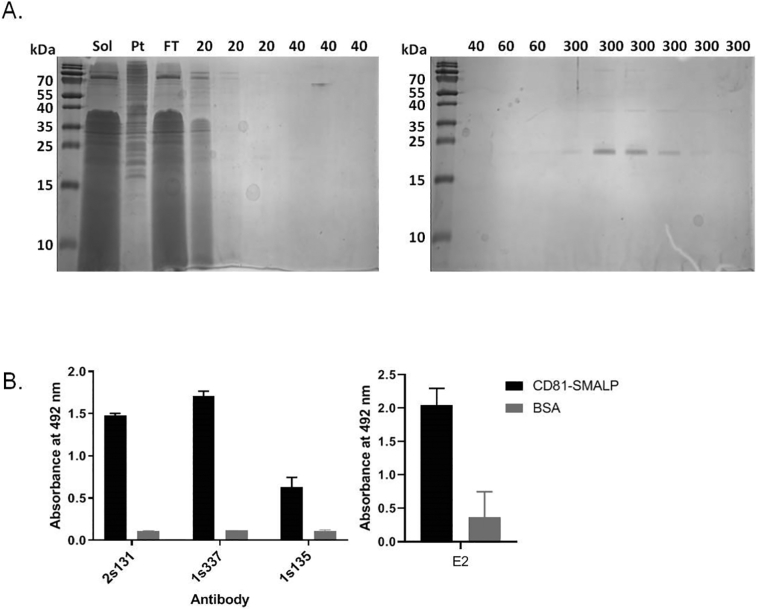
Purification of SMALP-encapsulated CD81 yields a functionally-folded protein. A) The Ni-NTA affinity purification procedure was modified to increase the amount of resin used, and increase the volume of washes. Samples of each step of the purification were analysed by SDS-PAGE stained with Instant Blue. Sol is the total solubilised protein, Pt is the insoluble protein, FT is flow-through and numbers show the concentration in mM of imidazole in the wash buffers. B) Elution fractions containing purified CD81 were pooled together and their folded state assessed by an ELISA using conformation-sensitive anti-CD81 antibodies; 1s337 and 1s135, alongside the linear peptide antibody 2s131. Bovine serum albumin was used as a negative control. Primary antibodies were detected with an anti-mouse-HRP secondary antibody and visualised by SIGMA*FAST*™ OPD tablet. Optical density at 492 nm was measured. Data are mean ± SD, n = 2. Purified CD81 was also analysed for binding to the E2 glycoprotein (that acts as a CD81 ligand) by ELISA , n = 3.

Having established that CD81 could be successfully solubilised by SMA 2000, the next step was to purify the protein. An initial Ni-NTA affinity purification (Supplementary Fig. 1) followed a published protocol for SMALPs [21]. While CD81-SMALP was found to bind well initially to the Ni^2+^ resin, with little protein in the flow through (Supplementary Fig. 1B), CD81 started to come off the column during the washes, and in the elution fractions it co-purified with a number of contaminants (Supplementary Fig. 1A). Mass spectrometry analysis of these contaminating bands identified plasma membrane H^+^ ATPase and alcohol dehydrogenase as particularly prominent contaminants (Supplementary Fig. 2). Therefore the purification procedure was optimized by i) increasing the amount of Ni-NTA resin used, which seemed to improve the binding of CD81 and ii) increasing the volume of washing steps to remove contaminating bands. This resulted in a much purer preparation of CD81 as shown in Fig. 3A. The structural integrity of this purified CD81 was tested by an ELISA assay using conformation sensitive anti-CD81 antibodies 1s337 and 1s135, which are known to recognize non-linear epitopes within the large extracellular loop of CD81, alongside an antibody which recognizes a linear peptide sequence 2s131 [22], as well as binding to the hepatitis C virus (HCV) E2 glycoprotein. As can be seen in Fig. 3B, all three antibodies and the E2 glycoprotein bound well to the purified CD81, showing that the purified CD81 retained its folded structure. For downstream comparisons, CD81 was also purified using DDM at comparable yields (Supplementary Table 2), and this CD81-DDM also bound the conformation sensitive antibodies and HCV E2 glycoprotein (Supplementary Fig. 3).

### 3.3 Biophysical comparison of CD81-SMALP and CD81-DDM

**Fig. 4:**
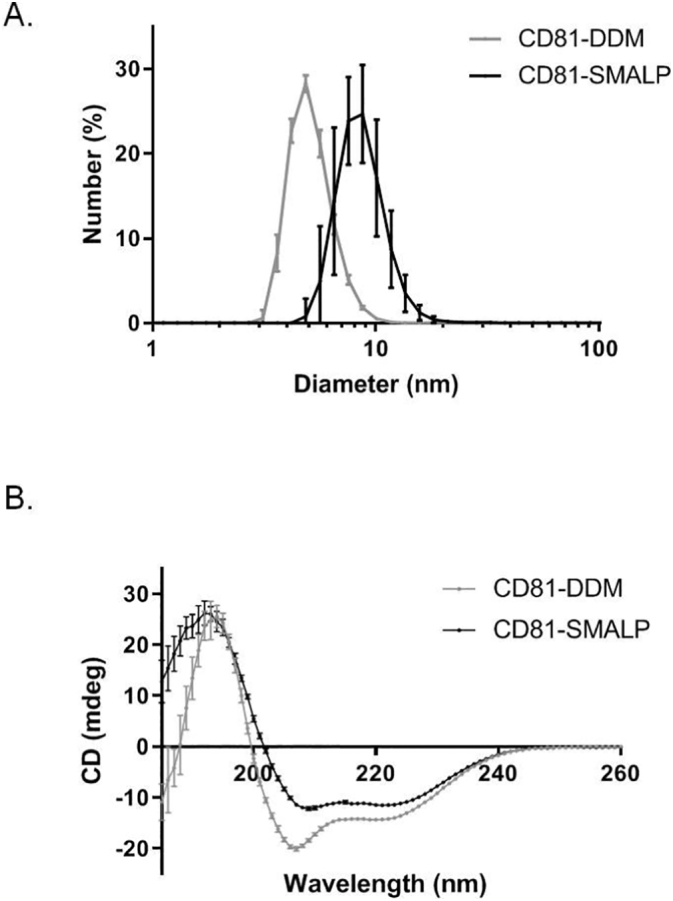
Biophysical characterisation of purified CD81. A) DLS analysis of purified CD81 either encapsulated within SMALPs (black) or within DDM micelles (grey) obtained using a Malvern Instruments Zetasizer Nano S (633 nm) with a 1.0 cm path length disposable cuvette. Seven technical replicates were performed for each sample, n = 2. B) CD spectra of purified CD81 either encapsulated within SMALPs (black) or within DDM micelles (grey). Purified CD81-SMALP and CD81-DDM was buffer exchanged into 20 mM sodium phosphate buffer pH 8 at concentrations of 0.05 mg/ml and 0.09 mg/ml, respectively. 200 μl of each sample was used in a 1 mm path length cuvette and measured using a Jasco J-1500 instrument. Maximum absorbance of 260 nm and minimum absorbance of 180 nm was used for CD detection at 0.2 nm intervals at 20 °C; 18 technical replicates were performed for each sample, where n = 2.

The biophysical properties of purified CD81-SMALP were then analysed and compared to those of CD81-DDM. Dynamic light scattering (DLS) was used to investigate the size of the particles. CD81-SMALPs had a diameter of approximately 10 nm which agrees well with previously reported studies using SMALPs [36,39]. CD81-DDM particles were smaller at approximately 5 nm, but this also agrees well with previously reported DDM micelle size [36]. To examine the folding of the protein, CD spectroscopy was used. It should be noted that for this CD spectroscopy analysis, CD81 samples were exchanged into a phosphate buffer lacking glycerol and NaCl, because of high light absorbance of Cl^−^ ions in the UV range. Fig. 4 shows that both CD81-SMALP and CD81-DDM display two negative peaks at 222 nm and 208 nm and a positive peak at 190 nm in the CD spectrum. This suggests that protein in both samples is folded and abundant in alpha-helical secondary structure. However, there is a clear difference in the shape of the CD81-DDM curve compared to CD81-SMALP, particularly at the 208 nm peak. After Dichroweb CD data analysis and taking the average alpha helical abundance from three secondary structure databases (CDSSTR, Contin-LL and Selcon3 – Supplementary Table 1), the CD81-SMALP appears to comprise 71% alpha helical structure and around 28% of the protein was classified as unstructured. This is in contrast to CD81-DDM where approximately 58% of the protein was alpha helical and 37% protein was classified as unstructured. This suggests that SMA solubilised and purified CD81 was better able to retain its correct structural conformation, since a previously-published CD81 crystal structure (PDB ID: 5TCX) indicated that approximately 68% of the protein is in alpha helical secondary structure [7].

**Fig. 5:**
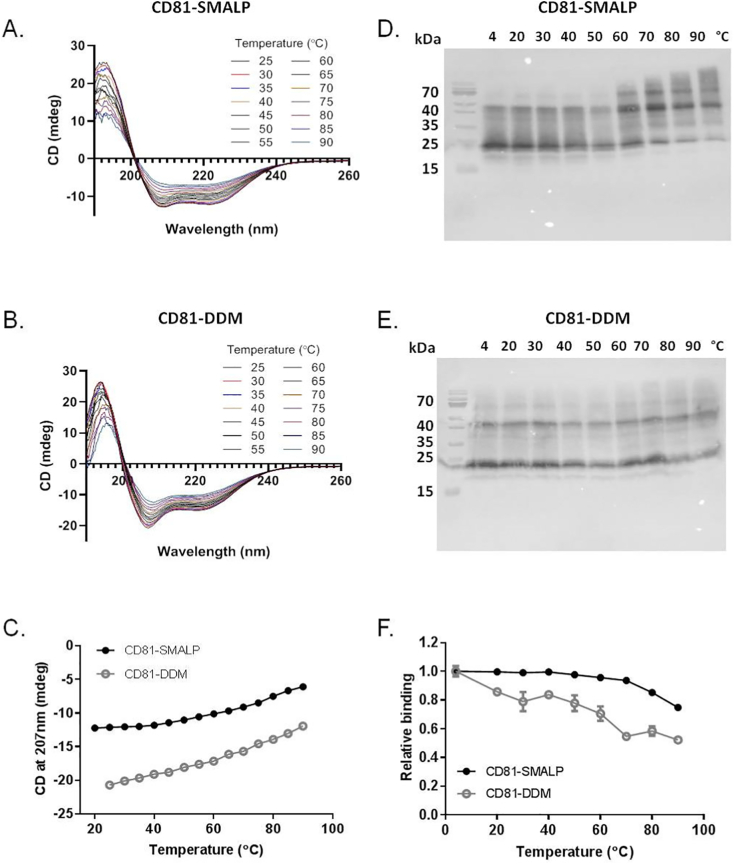
Thermostability of purified CD81. Effect of temperature on secondary structure measured by CD spectroscopy over a range of different temperatures for A) CD81-SMALP and B) CD81-DDM. Temperatures ranged from 25 °C to 90 °Cin 5 °C increments. Eighteen technical replicates were taken at each condition, n = 2. C) The temperature dependenceof the CD signal at 207 nm for both CD81-SMALP (black closed circles) and CD81-DDM (grey open circles). D & E) Effect of temperature on protein aggregation analysed by Western blot using an anti-CD81 antibody (mAb 2s131) for D) CD81-SMALP and E) CD81-DDM. F) Effect of increasing temperature on the binding of CD81 to a conformation sensitive anti-CD81 (mAb 1s337) antibody, using an ELISA. Black solid circles are CD81-SMALP, open grey squares are CD81-DDM. Data are mean ± SEM, n = 3.

The thermostability of purified CD81 was investigated next, using three different approaches. Firstly, CD spectroscopy was used to monitor protein secondary structure stability over a temperature range from 20 °C to 90 °C (Fig. 5A & B). At temperatures up to 40 °C, minor unfolding was observed, with CD81-SMALP possibly being a little more stable than CD81-DDM. However at temperatures above 40 °C the ellipticity reduced at each temperature increment. Despite the initial spectral differences observed between CD81-SMALP and CD81-DDM (Fig. 4B), little difference was seen between them in resisting heat treatment (Fig. 5C). The second approach examined protein aggregation upon heating (Fig. 5D & E). After heating, samples were centrifuged to remove large aggregates before visualising by Western blot. CD81-SMALP appeared unaffected up to temperatures of 40–50 °C. However at temperatures of 60 °C and higher, larger molecular weight aggregates are visible (Fig. 5D). In contrast CD81-DDM showed little variation even at high temperatures (Fig. 5E). Finally thermostability was monitored by binding to a conformationally sensitive antibody (Fig. 5F). CD81-SMALP showed very little change in binding at increasing temperatures up until 60 °C, suggesting intact conformation of CD81. At temperatures of 70 °C and above, a gradual decrease in antibody binding was observed, likely due to heat-induced unfolding of the extracellular loop 2 epitope. In contrast CD81-DDM antibody binding decreased following incubation at 20 °C and continued to decrease at each increased temperature, indicating lower protein stability in detergent micelles at this important extracellular loop than when CD81 is encapsulated in SMALPs. Both the aggregation and antibody binding assays utilized CD81-DDM in its optimized buffer composition containing both glycerol and NaCl.

### 3.4 Size exclusion chromatography of CD81-SMALP

**Fig. 6:**
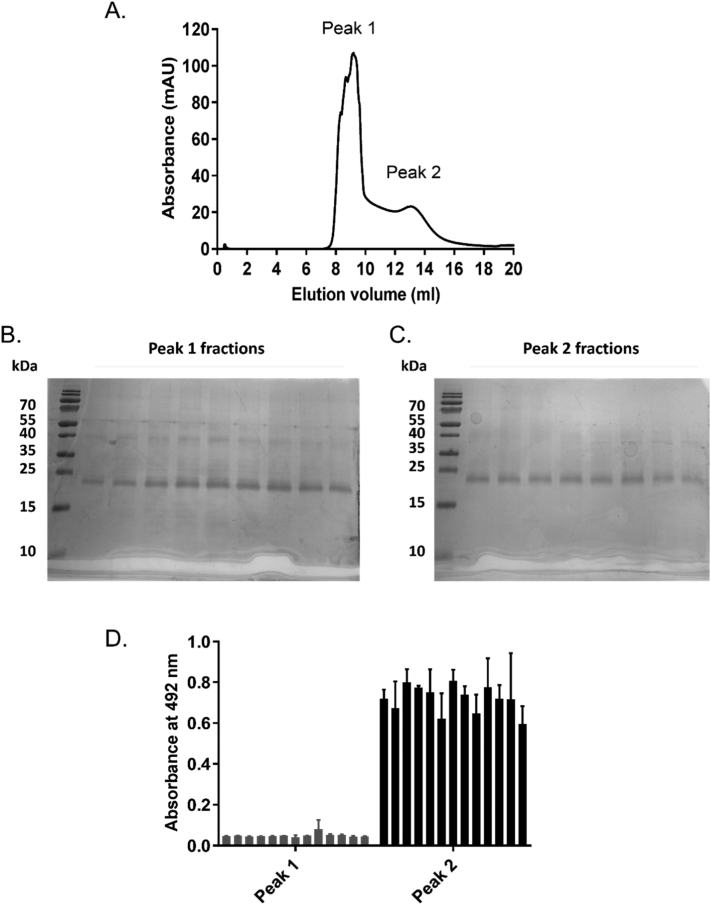
Size exclusion chromatography (SEC) analysis of CD81-SMALP reveals two distinct protein populations. A) Following Ni-NTA affinity chromatography, elution fractions were pooled, concentrated and 500 μl at 1 mg/ml were loaded on a Superdex 200 30/10 size exclusion column at 0.5 ml/min. Protein absorbance was monitored at 280 nm. B & C) Fractions from the two main peaks obtained by SEC were run on SDS-PAGE and stained with Instant Blue. D) Fractions from the two SEC peaks were analysed for binding to the E2 glycoprotein (that acts as CD81 ligand) by ELISA, n = 2.

Purified CD81-SMALP was concentrated and loaded on a size exclusion chromatography column. As shown in Fig. 6A, this resulted in two major elution peaks as measured by the absorbance at 280 nm. Peak 1 was very close to the void volume of the column (≈8 ml) suggesting the presence of larger complexes or SMALP aggregates. Peak 2 eluted at ≈13 ml column elution volume suggesting extraction of smaller molecules (potential isolation of individual CD81 SMALPs) in this region. When these fractions were analysed by SDS-PAGE (Fig. 6B), it was clear that both contained CD81, at fairly similar concentrations, although Peak 1 also contained higher molecular weight bands. Interestingly, when fractions from both peaks were assayed for their ability to bind the HCV E2 glycoprotein, only those from peak 2 showed binding (Fig. 1D).

**Fig. 7:**
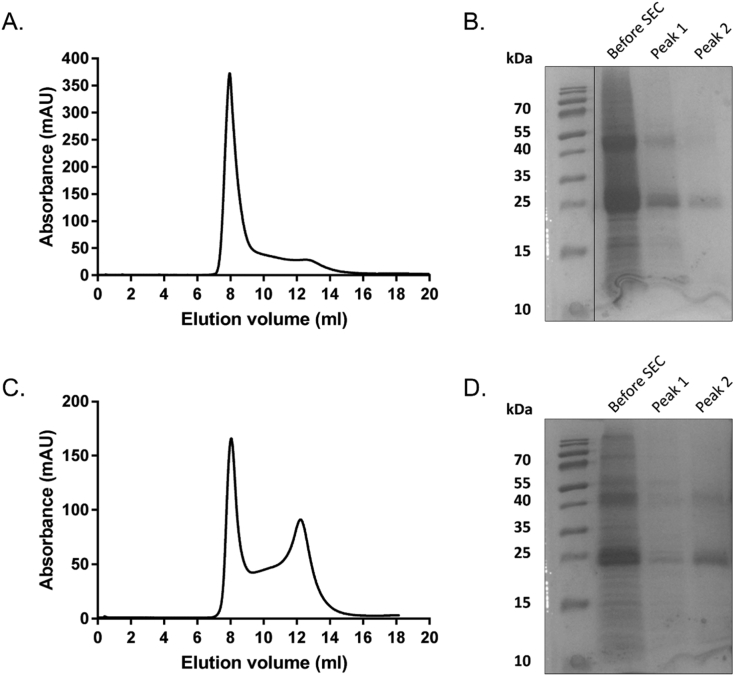
Optimization of SEC conditions increases the proportion of E2-binding-competent CD81. A) SEC profile of purified CD81-SMALP obtained using standard conditions. B) SDS-PAGE of the CD81 sample loaded on the column, peak 1 and peak 2 from the SEC trace in A. C) SEC profile of purified CD81-SMALP obtained using modified expression and buffer conditions. D) SDS-PAGE of the CD81 sample loaded on the column, peak 1 and peak 2 from the SEC trace in C. Traces are representative of those seen in 3 repeats.

As Peak 2 on the gel filtration trace appeared to contain functional, non-aggregated CD81, efforts were made to try to increase the proportion of CD81 in this peak. Consequently *P. pastoris* growth conditions were changed: lower cell biomass was taken forward for large scale growth in methanol medium (OD_600_ = 1) instead of inducing expression at high cell density (OD_600_ ˃ 5). Although this produced less CD81 (0.43 mg/l culture) than the standard higher biomass protocol (1.06 mg/l culture), a higher propoprtion was seen in peak 2 (Fig. 7 & Supplementary Table 2). Having identified that this change improved the quality of the protein, further optimization steps were made. HEPES buffer was used for solubilisation, purification and gel filtration work, supplemented with 200 mM sodium chloride and 10% glycerol, instead of the standard Tris/sodium chloride buffer. HEPES is often considered to buffer hydrogen ions more effectively than Tris, thus preventing pH-related SMALP or protein aggregation (although increasing the concentration of Tris could have been another approach), and glycerol is often reported to aid stability of membrane proteins. Finally, CD81-SMALP elution fractions from affinity purification were concentrated less before analysing on the SEC to minimise aggregation due to heavy concentration of the protein. A like-for-like comparison of the results obtained before and after optimisation are shown in Fig. 7. After applying these changes, an increase in Peak 2 was observed in the SEC spectrum (Fig. 7C). SDS-PAGE analysis of the SEC fractions shows considerably less protein in the aggregated fractions (Peak 1) and more in ≈13 ml elution fractions (Peak 2) (Fig. 7D).

### 3.5 Biophysical characterisation of the two size exclusion peaks

**Fig. 8:**
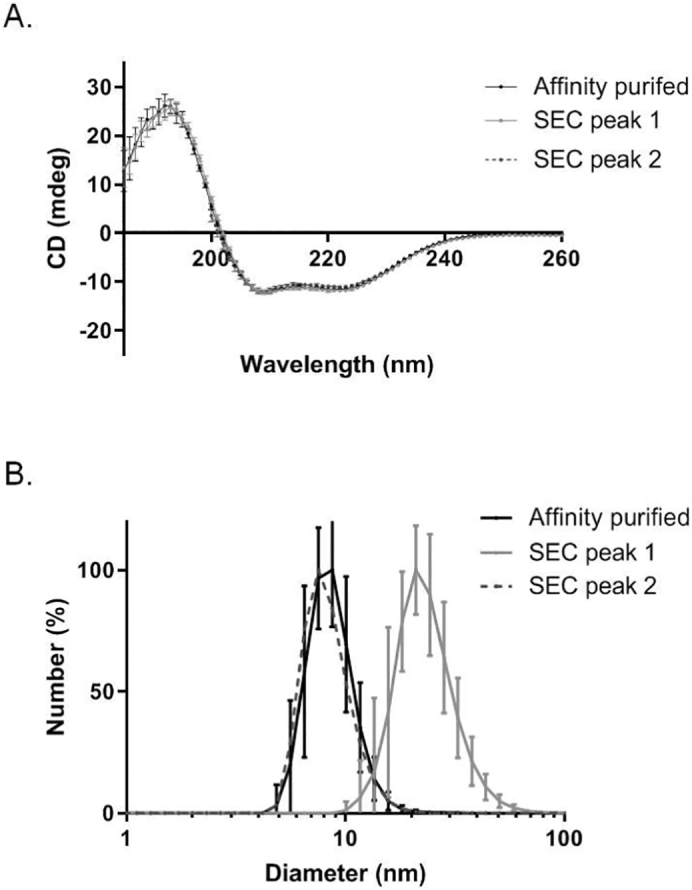
Biophysical characterisation of CD81–SMALP following size exclusion chromatography. A) CD spectra of affinity purified CD81-SMALP prior to SEC (black), CD81-SMALP in peak 1 following SEC (light grey) and from peak 2 following SEC (dark grey, dashed). 200 μl of each sample was used in a 1 mm path length cuvette and measured using a Jasco J-1500 instrument. Maximum absorbance of 260 nm and minimum absorbance of 180 nm was used for CD detection at 0.2 nm intervals at 20 °C; 18 technical replicates were performed for each sample, where n = 2. B) DLS analysis of purified CD81-SMALP prior to SEC (black), CD81-SMALP in peak 1 following SEC (light grey) and from peak 2 following SEC (dark grey, dashed). Data were obtained using a Malvern Instruments Zetasizer Nano S (633 nm) with 1.0 cm path length disposable cuvette. Seven technical replicates were performed for each sample, n = 2.

To further investigate the nature of the proteins present in each of the two peaks observed with SEC, the two samples were characterized by DLS, CD and EM. CD spectra of both peaks from SEC overlaid perfectly with the sample straight off the affinity purification column, showing that both peak 1 and peak 2 contained fully folded CD81 (Fig. 8A). However DLS analysis of the same samples showed that the particles within peak 1 were larger than those within peak 2, with an average diameter of approximately 20 nm. When examined by negative stain EM, the particles within peak 1 also appeared larger than those in peak 2 (Supplementary Fig. 4).

### 3.6 Analysis of CD81 lipid environment

**Fig. 9:**
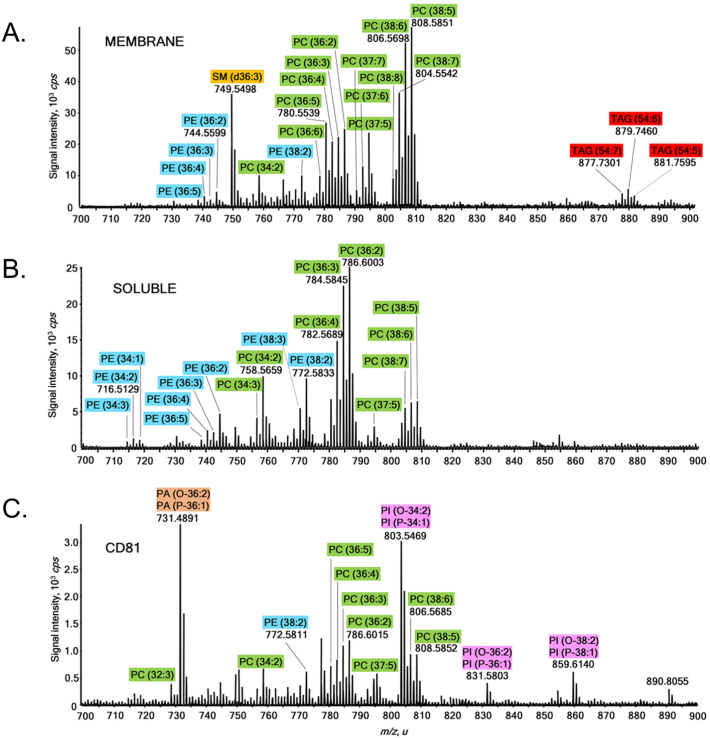
Positive ion mode MS spectra measured for total lipid extracts of total yeast membrane, SMA solubilised membrane and purified CD81 SMALP. Identified glycerophosphatidylcholines are labelled in green (PC), glycerophosphatidyletanolamines in blue (PE), glycerophosphatidylinositols in magenta (PI), glycerophosphatidycacids in peach (PA) and sphingomyelin in orange (SM). (For interpretation of the references to colour in this figure legend, the reader is referred to the web version of this article.)

**Fig. 10:**
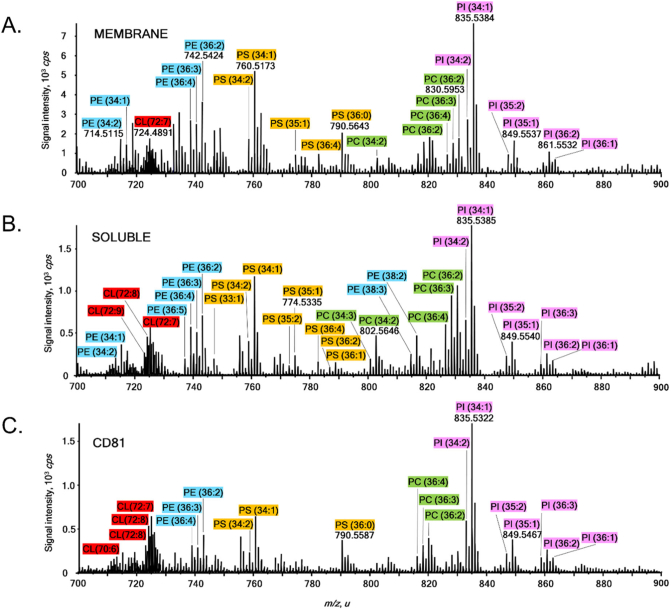
Negative ion mode MS spectra measured for total lipid extracts of total yeast membrane, SMA solubilised membrane and purified CD81 SMALP. Identified glycerophosphatidylcholines are labelled in green (PC), glycerophosphatidyletanolamines in blue (PE), glycerophosphatidylinositols in magenta (PI), glycerophosphatidylserines in orange (PS) and cardiolipins in red (CL). (For interpretation of the references to colour in this figure legend, the reader is referred to the web version of this article.)

As SMALPs extract membrane proteins within their lipid environment, we used a shotgun lipidomics approach to investigate changes in the lipid environment of purified CD81-SMALP. We compared the lipid profiles of total membrane extracts from *P. pastoris* overexpressing CD81 (Figs. 9A and 10A), the SMA solubilised membranes (Figs. 9B and 10B) and purified CD81-SMALPs (Figs. 9C and 10C). In total, we were able to detect lipids across eight different lipids classes, namely phosphatidylcholines, phosphatidylethanolamines, phosphatidycacids, phosphatidylserines, phosphatidylinositols, triacylglycerols, sphingomyelin and cardiolipins.

The positive ion mode mass spectrum of total membranes (Fig. 9A) is dominated by phosphatidylcholine species. The phosphatidylcholine lipids have relatively long polyunsaturated fatty acid chains of 38 carbon atoms and five to eight double bonds and phosphatidylcholine (38:5) appears as the most intense lipid ion. Phosphatidylethanolamine lipids were detected with significantly lower signal intensities, lower complexity, shorter polyunsaturated fatty acid chains and lower unsaturation levels. A single sphingomyelin species, sphingomyelin (d36:3) was observed with relatively strong signal intensity. Finally, a few lipids from a triacylglycerol family, eg triacylglycerol (54:6) were also detected. Negative ion mode spectra (Fig. 10A) suggest phosphatidylinositol is the most dominant lipid class detected. Phosphatidylinositol lipids were detected with polyunsaturated fatty acid chains of predominantly 34C atoms and up to two double bonds. Phosphatidylserine and cardiolipin were also observed in the total membrane extracts of *P. pastoris* overexpressing CD81.

Mass spectra of SMA solubilised membranes revealed some differences in measured lipid profiles compared to the total membranes. We observed the total loss of sphingomyelin and triacylglycerol lipids (Fig. 9B). Although there was no change in the overall complexity of phosphatidylcholine lipids, it is apparent that phosphatidylcholine lipids with shorter polyunsaturated fatty acid chains and lower unsaturation levels, e.g. phosphatidylcholine (36:2) were now the most dominant ones. Phosphatidylethanolamine and phosphatidylserine profiles appear more diverse in SMA-solubilised membranes compared to total membranes. However, the complexity and relative intensities of negatively-charged lipids, in particular phosphatidylinositol and cardiolipin stayed unaffected by the SMA membrane solubilisation.

Significant changes in phosphatidylcholine and PE species were observed upon purification of CD81-SMALP from SMA solubilised membranes. Although phosphatidylcholine lipids with reduced complexity were still present, they were no longer the dominant species in the positive ion mode, while PE lipids were almost absent (Fig. 9C). Surprisingly, phosphatidycacid and phosphatidylinositol lipids dominated even the positive ion spectrum of CD81-SMALP. A single phosphatidycacid lipid, namely phosphatidycacid (O-36:2)/phosphatidycacid (P-36:1) was detected as the most intense signal in a form of an alkyl ether. Corresponding tandem mass spectrum showed the prominent neutral losses of water and CO_2_, characteristic of carboxylic acids. Similarly, a phosphatidylinositol alkyether lipid such as phosphatidylinositol (O-34:2)/phosphatidylinositol (P-34:1) was detected as the second most dominant signal. The negative ion mode measurements revealed that phosphatidylinositol and cardiolipin diversity and relative abundances remained conserved even upon purification of CD81-SMALP, while phosphatidylserine heterogeneity was reduced.

## 4 Discussion

In this study we have shown that recombinant CD81 expressed in *P. pastoris* yeast can be effectively solubilised and purified using SMA polymer. It was previously shown that another member of the tetraspanin family, TSPAN7, could also be extracted with SMA from the yeast *Sacchromyces cerevisiae*. However this was not the case for several other tetraspanins expressed in *S. cerevisiae* [40]. Interestingly, we found that monitoring membrane turbidity was not a good measure of CD81 solubilisation. Membrane turbidity is quick and easy to measure and has been used previously to monitor kinetics of solubilisation [37,41]. However we found that turbidity decreased much more quickly and at lower concentrations of SMA than were needed for CD81 solubilisation (Fig. 2). This contrasts with a previous report where SMA was used to solubilise the ABC transporter MRP4/ABBC4 (multidrug resistance protein 4) from insect cell membranes, where the changes in turbidity were mirrored by protein specific solubilisation [42]. Secondly the speed of CD81 solubilisation was much slower than that observed for MRP4 [42], possibly related to the differences in lipid composition of yeast membranes compared to insect cells [43]. Thus this highlights the importance of measuring protein-specific solubilisation efficiency and that optimal conditions will likely be both protein and expression-system dependent.

As has been observed previously for many other proteins, purification of CD81 using SMA showed comparable yields to that obtained using conventional detergents but gave a cleaner purification [17,36,44]. CD spectroscopy also showed subtle differences between SMA and DDM purified CD81, suggesting that SMA encapsulated CD81 retained more of the native secondary structure. It should be noted that this was a side-by-side comparison under comparable conditions, in buffer that did not contain NaCl or glycerol, which have been found to stabilize the DDM purified protein. This may explain why the DDM sample was less folded than the previously published crystal structure which was also obtained using DDM [7]. However it highlights that SMALPs can offer improved stability without the need for special handling or buffer conditions. Interestingly, SMA did not offer any improvement over DDM for CD81 thermostability as measured by CD spectroscopy or when using a gel based aggregation assay, which contrasts with many previous reports [13,36,44]. The reasons for this are not totally clear; perhaps the secondary structure of CD81 is inherently quite stable, and it has been suggested previously that the presence of detergent can help to decrease aggregation for some proteins [36]. Differences between DDM and SMA purified CD81 were observed when using a binding assay for a conformation-sensitive antibody. This antibody is known to bind to a non-linear epitope within the large extracellular loop, which is important for function of CD81 [22]. Thus SMA encapsulation stabilised the 3-dimensional folded structure of CD81, even if very little change in secondary structure occurred. Notably this antibody binding assay utilized CD81-DDM within its optimized buffer conditions containing both glycerol and NaCl, so this difference is not simply caused by using suboptimal conditions for the detergent solubilised protein.

SEC analysis of SMA purified CD81 showed a large peak in or close to the void volume in addition to a second peak that eluted later. Presumably the void peak contains some form of larger assembly or aggregated species, however it is notable that both peaks contain fully folded CD81, thus the void peak does not contain denatured, aggregated protein. CD81 itself is known to form dimers and larger assemblies [4,5] so it is possible that this peak contains larger assemblies of CD81. Notably, the CD81 crystal structure revealed a monomeric form of a tetraspanin, which contrasts with the crystal structure of the extracellular LEL domain of human CD81 (88 of 236 residues). This latter structure confirms the presence of the conserved CCG motif, two disulfide bridges and a potential dimerisation interface [45]. The DLS analysis of the void peak shows that it is approximately twice the size of a standard SMALP. One possibility is that CD81 has oligomerised and the SMALP formed around the oligomer is larger to accomodate this. Such flexibility of size has been observed previously [46]. Alternatively it has also been shown previously that SMALP discs themselves can dimerise [47]. It was interesting that the intensity of the A_280_ signal for the void peak was significantly higher than that of the second peak, and yet SDS-PAGE analysis showed almost equal concentrations of CD81 in both peaks. Therefore perhaps the species present in the void peak was also scattering light. Importantly only the CD81 in the second peak bound to the HCV E2 glycoprotein. Given that CD spectroscopy showed the CD81 in the void peak was fully folded, it is unlikely that the protein has become sufficiently unstructured such that the E2 binding site is not formed. It is more likely that access to the binding site is sterically blocked in some way. Whether this is due to oligomerisation of CD81 itself, or of the SMALP complex, or caused by a conformational change of the protein remains to be determined. Going forward it will be interesting to investigate in more detail the nature of the CD81-polymer complexes in the two peaks, especially given that the proportion of CD81 present in the second peak could be improved by changing expression and buffer conditions.

Lastly, we examined the lipids associated with SMA-purified CD81. Importantly, we first analysed the lipid species present in whole membranes from *P. pastoris* expressing CD81, and SMA solubilised *P. pastoris* membranes. In total, lipids from seven families were detected in total membrane extracts, namely phosphatidylcholine, phosphatidylethanolamine, sphingomyelin, triacylglycerol, phosphatidylserine, phosphatidylinositol and cardiolipin. These results are in good agreement with previously published work [48,49]. Ejsing et al. investigated the total lipidome of *S. cerevisiae* in one of the most comprehensive yeast lipid profiling studies published to date [48]. Although here we report the membrane lipidome of *P. pastoris*, we have detected the presence of lipids from the same lipid classes as observed with *S. cerevisiae* [48]. Furthermore, it appears that in both *P. pastoris* and *S. cerevisiae* phosphatidylcholine, phosphatidylethanolamine and phosphatidylinositol lipids are the most dominant ones. Grillitsch et al. reported that the most abundant phospholipid class detected in *P. pastoris* plasma membranes was phosphatidylethanolamine, followed by phosphatidylcholine, phosphatidylserine, phosphatidycacid, phosphatidylinositol and cardiolipin [49]. Our results detected no phosphatidyglycerol lipids in the *P. pastoris* membrane, in agreement with both previous studies in yeast [48,49]. Further, sterol lipids, primarily ergosterol as one of the major constituents of yeast membranes, were not identified. Its abundance is decreased up to 8-fold in *P. pastoris* compared to *S. cerevisiae* [50]. Thus, future work should include the design of more targeted lipidomics approaches for the detection of sterols. It is important to note that the lipid fingerprint of yeast membranes depends on growth conditions such as temperature, carbon source and oxygen supply, and these could contribute to the quantitative difference observed between studies [[51], [52], [53]]. Furthermore, it was previously shown that SMA solubilisation is not preferential for any particular lipid subset [[13], [14], [15],^54^]. Nevertheless, in SMA-solubilised membranes we have observed loss of the signals for sphingomyelin (d36:3) and triacylglycerol, higher diversity of phosphatidylethanolamine and phosphatidylserine lipids, and the change in signal intensities of phosphatidylcholine lipids while their heterogeneity remained the same. The most dramatic change in lipid fingerprint was observed in CD81-SMALP. This was reflected in an apparent decrease in positively charged phosphatidylethanolamine and phosphatidylserine lipids, and enrichment in negatively charged phosphatidylserine, phosphatidycacid and phosphatidylinositol. Both phosphatidylserine and phosphatidylinositol play a crucial role in generating a negative surface plasma membrane potential that is essential for lipid binding of proteins with pleckstrin homology domains [55]. Phosphatidylinositol lipids are present in relatively low abundance in cells and tissues, but in a high concentration in plasma membranes. They serve as precursors of several signalling lipids and play a crucial role in signal transduction. Carloni et al. showed that transfection of human cells with CD81 stimulated phosphatidylinositol-4-kinase activity at the plasma membrane, increasing the levels of phosphatidylinositol-4-phosphate which affected protein recruitment, cellular signalling and proliferation [56].

It should be noted that CD81 is a human protein that was expressed recombinantly in *P. pastoris*, so it is not within a fully-native lipid environment. Going forward, it would be of interest to investigate whether similar lipid profiles are observed with CD81 expressed in mammalian cells. It would also be interesting to reconstitute purified CD81 into liposomes of defined lipids, as identified in this study, and examine the effect of the lipids on CD81 function, structure and stability. Our findings set the scene for understanding the role of protein-lipid interactions in the biology of CD81 and of the tetraspanin family more broadly.

## Credit author contribution

**Hoor Ayub**: Conceptualization, Methodology, Validation, Analysis, Investigation, Writing – original draft. **Michelle Clare**: Methodology, Investigation, Writing – review & editing. **Ivana Milic**: Analysis, Investigation, Resources, Writing – original draft. **Nikola P. Chmel**: Analysis, Investigation, Resources, Writing – review & editing. **Heike Boning**: Resources, Investigation. **Andrew Devitt**: Supervision, Funding Acquisition, Writing – review & editing. **Thomas Krey**: Resources, Supervision, Funding Acquisition, Writing – review & editing. **Roslyn M. Bill**: Conceptualization, Resources, Supervision, Funding Acquisition, Writing – original draft. **Alice J. Rothnie**: Conceptualization, Methodology, Supervision, Writing – original draft.

## Declaration of competing interest

The authors declare that they have no known competing financial interests or personal relationships that could have appeared to influence the work reported in this paper.

## Appendix A Supplementary data

Supplementary material Image 1

## References

[1] Hemler M.E. (2005). Tetraspanin functions and associated microdomains. Nat. Rev. Mol. Cell Biol..

[2] Hemler M.E. (2008). Targeting of tetraspanin proteins-potential benefits and strategies. Nat. Rev. Drug Discov..

[3] Hemler M.E. (2014). Tetraspanin proteins promote multiple cancer stages. Nat. Rev. Cancer.

[4] Bonander N., Jamshad M., Oberthur D., Clare M., Barwell J., Hu K., Farquhar M.J., Stamataki Z., Harris H.J., Dierks K., Dafforn T.R., Betzel C., McKeating J.A., Bill R.M. (2013). Production, purification and characterization of recombinant, full-length human claudin-1. PLoS One.

[5] Kovalenko O.V., Yang X., Kolesnikova T.V., Hemler M.E. (2004). Evidence for specific tetraspanin homodimers: inhibition of palmitoylation makes cysteine residues available for cross-linking. The Biochemical Journal.

[6] Min G., Wang H., Sun T.T., Kong X.P. (2006). Structural basis for tetraspanin functions as revealed by the cryo-EM structure of uroplakin complexes at 6-A resolution. J. Cell Biol..

[7] Zimmerman B., Kelly B., McMillan B.J., Seegar T.C.M., Dror R.O., Kruse A.C., Blacklow S.C. (2016). Crystal structure of a full-length human tetraspanin reveals a cholesterol-binding pocket. Cell.

[8] Umeda R., Satouh Y., Takemoto M., Nakada-Nakura Y., Liu K., Yokoyama T., Shirouzu M., Iwata S., Nomura N., Sato K., Ikawa M., Nishizawa T., Nureki O. (2020). Structural insights into tetraspanin CD9 function. Nat. Commun..

[9] Feneant L., Levy S., Cocquerel L. (2014). CD81 and hepatitis C virus (HCV) infection. Viruses.

[10] Dawaliby R., Trubbia C., Delporte C., Masureel M., Van Antwerpen P., Kobilka B.K., Govaerts C. (2016). Allosteric regulation of G protein-coupled receptor activity by phospholipids. Nat. Chem. Biol..

[11] Zoghbi M.E., Cooper R.S., Altenberg G.A. (2016). The lipid bilayer modulates the structure and function of an ATP-binding cassette exporter. J. Biol. Chem..

[12] Rothnie A., Theron D., Soceneantu L., Martin C., Traikia M., Berridge G., Higgins C.F., Devaux P.F., Callaghan R. (2001). The importance of cholesterol in maintenance of P-glycoprotein activity and its membrane perturbing influence. Eur. Biophys. J..

[13] Dorr J.M., Koorengevel M.C., Schafer M., Prokofyev A.V., Scheidelaar S., van der Cruijsen E.A., Dafforn T.R., Baldus M., Killian J.A. (2014). Detergent-free isolation, characterization, and functional reconstitution of a tetrameric K+ channel: the power of native nanodiscs. Proc. Natl. Acad. Sci. U. S. A..

[14] Teo A.C.K., Lee S.C., Pollock N.L., Stroud Z., Hall S., Thakker A., Pitt A.R., Dafforn T.R., Spickett C.M., Roper D.I. (2019). Analysis of SMALP co-extracted phospholipids shows distinct membrane environments for three classes of bacterial membrane protein. Sci. Rep..

[15] Prabudiansyah I., Kusters I., Caforio A., Driessen A.J. (2015). Characterization of the annular lipid shell of the sec translocon. Biochim. Biophys. Acta.

[16] Jamshad M., Grimard V., Idini I., Knowles T.J., Dowle M.R., Schofield N., Sridhar P., Lin Y.P., Finka R., Wheatley M., Thomas O.R.T., Palmer R.E., Overduin M., Govaerts C., Ruysschaert J.M., Edler K.J., Dafforn T.R. (2015). Structural analysis of a nanoparticle containing a lipid bilayer used for detergent-free extraction of membrane proteins. Nano Res..

[17] Gulati S., Jamshad M., Knowles T.J., Morrison K.A., Downing R., Cant N., Collins R., Koenderink J.B., Ford R.C., Overduin M., Kerr I.D., Dafforn T.R., Rothnie A.J. (2014). Detergent-free purification of ABC (ATP-binding-cassette) transporters. Biochem. J..

[18] Jamshad M., Charlton J., Lin Y.P., Routledge S.J., Bawa Z., Knowles T.J., Overduin M., Dekker N., Dafforn T.R., Bill R.M., Poyner D.R., Wheatley M. (2015). G-protein coupled receptor solubilization and purification for biophysical analysis and functional studies, in the total absence of detergent. Biosci. Rep..

[19] Pollock N.L., Lee S.C., Patel J.H., Gulamhussein A.A., Rothnie A.J. (2018). Structure and function of membrane proteins encapsulated in a polymer-bound lipid bilayer. Biochim. Biophys. Acta.

[20] Jamshad M., Rajesh S., Stamataki Z., McKeating J.A., Dafforn T., Overduin M., Bill R.M. (2008). Structural characterization of recombinant human CD81 produced in Pichia pastoris. Protein Expr. Purif..

[21] Rothnie A.J. (2016). Detergent-free membrane protein purification. Methods Mol. Biol..

[22] Grove J., Hu K., Farquhar M.J., Goodall M., Walker L., Jamshad M., Drummer H.E., Bill R.M., Balfe P., McKeating J.A. (2017). A new panel of epitope mapped monoclonal antibodies recognising the prototypical tetraspanin CD81. Wellcome Open Res.

[23] Rothnie A., Storm J., Campbell J., Linton K.J., Kerr I.D., Callaghan R. (2004). The topography of transmembrane segment six is altered during the catalytic cycle of P-glycoprotein. J. Biol. Chem..

[24] Krey T., d’Alayer J., Kikuti C.M., Saulnier A., Damier-Piolle L., Petitpas I., Johansson D.X., Tawar R.G., Baron B., Robert B., England P., Persson M.A., Martin A., Rey F.A. (2010). The disulfide bonds in glycoprotein E2 of hepatitis C virus reveal the tertiary organization of the molecule. PLoS Pathog..

[25] Tarr A.W., Lafaye P., Meredith L., Damier-Piolle L., Urbanowicz R.A., Meola A., Jestin J.L., Brown R.J., McKeating J.A., Rey F.A., Ball J.K., Krey T. (2013). An alpaca nanobody inhibits hepatitis C virus entry and cell-to-cell transmission. Hepatology.

[26] Whitmore L., Wallace B.A. (2004). DICHROWEB, an online server for protein secondary structure analyses from circular dichroism spectroscopic data. Nucleic Acids Res..

[27] Whitmore L., Wallace B.A. (2008). Protein secondary structure analyses from circular dichroism spectroscopy: methods and reference databases. Biopolymers.

[28] Sreerama N., Woody R.W. (1993). A self-consistent method for the analysis of protein secondary structure from circular dichroism. Anal. Biochem..

[29] Sreerama N., Venyaminov S.Y., Woody R.W. (1999). Estimation of the number of alpha-helical and beta-strand segments in proteins using circular dichroism spectroscopy. Protein Sci..

[30] Provencher S.W., Glockner J. (1981). Estimation of globular protein secondary structure from circular dichroism. Biochemistry.

[31] van Stokkum I.H., Spoelder H.J., Bloemendal M., van Grondelle R., Groen F.C. (1990). Estimation of protein secondary structure and error analysis from circular dichroism spectra. Anal. Biochem..

[32] Compton L.A., Johnson W.C. (1986). Analysis of protein circular dichroism spectra for secondary structure using a simple matrix multiplication. Anal. Biochem..

[33] Manavalan P., Johnson W.C. (1987). Variable selection method improves the prediction of protein secondary structure from circular dichroism spectra. Anal. Biochem.

[34] Sreerama N., Woody R.W. (2000). Estimation of protein secondary structure from circular dichroism spectra: comparison of CONTIN, SELCON, and CDSSTR methods with an expanded reference set. Anal. Biochem..

[35] Matyash V., Liebisch G., Kurzchalia T.V., Shevchenko A., Schwudke D. (2008). Lipid extraction by methyl-tert-butyl ether for high-throughput lipidomics. J. Lipid Res..

[36] Morrison K.A., Akram A., Mathews A., Khan Z.A., Patel J.H., Zhou C., Hardy D.J., Moore-Kelly C., Patel R., Odiba V., Knowles T.J., Javed M.U., Chmel N.P., Dafforn T.R., Rothnie A.J. (2016). Membrane protein extraction and purification using styrene-maleic acid (SMA) copolymer: effect of variations in polymer structure. Biochem. J..

[37] Bersch B., Dorr J.M., Hessel A., Killian J.A., Schanda P. (2017). Proton-detected solid-state NMR spectroscopy of a zinc diffusion facilitator protein in native nanodiscs. Angew Chem Int Ed Engl.

[38] Rehan S., Jaakola V.P. (2015). Expression, purification and functional characterization of human equilibrative nucleoside transporter subtype-1 (hENT1) protein from Sf9 insect cells. Protein Expr. Purif..

[39] Swainsbury D.J., Scheidelaar S., van Grondelle R., Killian J.A., Jones M.R. (2014). Bacterial reaction centers purified with styrene maleic acid copolymer retain native membrane functional properties and display enhanced stability. Angew Chem Int Ed Engl.

[40] Skaar K., Korza H.J., Tarry M., Sekyrova P., Hogbom M. (2015). Expression and subcellular distribution of GFP-tagged human tetraspanin proteins in Saccharomyces cerevisiae. PLoS One.

[41] Logez C., Damian M., Legros C., Dupre C., Guery M., Mary S., Wagner R., M’Kadmi C., Nosjean O., Fould B., Marie J., Fehrentz J.A., Martinez J., Ferry G., Boutin J.A., Baneres J.L. (2016). Detergent-free isolation of functional G protein-coupled receptors into nanometric lipid particles. Biochemistry.

[42] Hardy D., Bill R.M., Rothnie A.J., Jawhari A. (2019). Stabilization of human multidrug resistance protein 4 (MRP4/ABCC4) using novel solubilization agents. SLAS Discov.

[43] Dominguez Pardo J.J., Dorr J.M., Iyer A., Cox R.C., Scheidelaar S., Koorengevel M.C., Subramaniam V., Killian J.A. (2017). Solubilization of lipids and lipid phases by the styrene-maleic acid copolymer. Eur. Biophys. J..

[44] Liu Y., Moura E., Dorr J.M., Scheidelaar S., Heger M., Egmond M.R., Killian J.A., Mohammadi T., Breukink E. (2018). Bacillus subtilis MraY in detergent-free system of nanodiscs wrapped by styrene-maleic acid copolymers. PLoS One.

[45] Kitadokoro K., Ponassi M., Galli G., Petracca R., Falugi F., Grandi G., Bolognesi M. (2002). Subunit association and conformational flexibility in the head subdomain of human CD81 large extracellular loop. Biol. Chem..

[46] Sun C., Benlekbir S., Venkatakrishnan P., Wang Y., Hong S., Hosler J., Tajkhorshid E., Rubinstein J.L., Gennis R.B. (2018). Structure of the alternative complex III in a supercomplex with cytochrome oxidase. Nature.

[47] Postis V., Rawson S., Mitchell J.K., Lee S.C., Parslow R.A., Dafforn T.R., Baldwin S.A., Muench S.P. (2015). The use of SMALPs as a novel membrane protein scaffold for structure study by negative stain electron microscopy. Biochim. Biophys. Acta.

[48] Ejsing C.S., Sampaio J.L., Surendranath V., Duchoslav E., Ekroos K., Klemm R.W., Simons K., Shevchenko A. (2009). Global analysis of the yeast lipidome by quantitative shotgun mass spectrometry. Proc. Natl. Acad. Sci..

[49] Grillitsch K., Tarazona P., Klug L., Wriessnegger T., Zellnig G., Leitner E., Feussner I., Daum G. (2014). Isolation and characterization of the plasma membrane from the yeast Pichia pastoris. Biochim. Biophys. Acta Biomembr..

[50] Zinser E., Paltauf F., Daum G. (1993). Sterol composition of yeast organelle membranes and subcellular distribution of enzymes involved in sterol metabolism. J. Bacteriol..

[51] Galea A.M., Brown A.J. (2009). Special relationship between sterols and oxygen: were sterols an adaptation to aerobic life?. Free Radic. Biol. Med..

[52] Schuiki I., Schnabl M., Czabany T., Hrastnik C., Daum G. (2010). Phosphatidylethanolamine synthesized by four different pathways is supplied to the plasma membrane of the yeast Saccharomyces cerevisiae. Biochim. Biophys. Acta.

[53] Inan M., Meagher M.M. (2001). Non-repressing carbon sources for alcohol oxidase (AOX1) promoter of Pichia pastoris. J. Biosci. Bioeng..

[54] Smirnova I.A., Sjostrand D., Li F., Bjorck M., Schafer J., Ostbye H., Hogbom M., Ballmoos C. von, Lander G.C., Adelroth P., Brzezinski P. (2016). Isolation of yeast complex IV in native lipid nanodiscs. Biochim. Biophys. Acta.

[55] Stahelin R.V. (2009). Lipid binding domains: more than simple lipid effectors. J. Lipid Res..

[56] Carloni V., Mazzocca A., Ravichandran K.S. (2004). Tetraspanin CD81 is linked to ERK/MAPKinase signaling by Shc in liver tumor cells. Oncogene.

