# Supplementary material for: CD81 extracted in SMALP nanodiscs comprises two distinct protein populations within a lipid environment enriched with negatively charged headgroups
Source: Biochim Biophys Acta Biomembr. 2020 Nov 1;1862(11):183419. doi: 10.1016/j.bbamem.2020.183419 (PMC7456796; doi:10.1016/j.bbamem.2020.183419)
Supplement: Supplementary file 1 — Supplementary material [file mmc1.docx]

**Supplementary Information**

**CD81 extracted in SMALP nanodiscs comprises two distinct protein populations within a lipid environment enriched with negatively charged headgroups**

Hoor Ayub, Michelle Clare, Ivana Milic, Nikola P. Chmel, Heike Böning, Andrew Devitt, Thomas Krey, Roslyn M. Bill & Alice J. Rothnie

**Supplementary Table 1**

| **Sample** | **Concentration (mg/ml)** | **Analysis** | **Secondary structure** | | | |
| --- | --- | --- | --- | --- | --- | --- |
|  |  |  | **α-helices** | **β-sheets** | **other** | **total** |
| **CD81-SMALP** | 0.05 | CDSSTR | 75 | 6 | 18 | 99 |
|  |  | CONTIN | 65 | 3 | 32 | 100 |
|  |  | SELCON3 | 74 | -4 | 34 | 104 |
|  |  | **average** | **71** | **2** | **28** | **100** |
|  |  |  |  |  |  |  |
|  |  |  |  |  |  |  |
| **CD81-DDM** | 0.09 | CDSSTR | 64 | 9 | 30 | 100 |
|  |  | CONTIN | 54 | 6 | 40 | 100 |
|  |  | SELCON3 | 59 | 3 | 40 | 102 |
|  |  | **average** | **58** | **6** | **37** | **101** |
|  |  |  |  |  |  |  |

**Supplementary Table 1: Parameters from Dichroweb analysis of CD spectroscopy data.** Baseline corrected data were analysed with Dichroweb using the CDSSTR, Contin-LL and Selcon3 algorithms. A mean weight residue of 109.8 was used. For SELCON3 analysis of CD81-SMALP, the data were truncated from 190nm. For all CD81-DDM analyses, data were truncated from 185nm to avoid the increase in CD due to absorbance from the detergent.

**Supplementary Table 2**

|  | **OD_600_ > 5** | | **OD_600_ = 1** | |
| --- | --- | --- | --- | --- |
| **Yeast cell pellet (g/l culture)** | 16.8 ± 0.3 | | 11.4 ± 0.1 | |
| **Membrane pellet (g/l culture)** | 1.63 ± 0.38 | | 1.08 ± 0.23 | |
| **Affinity purified protein (mg/l culture)** | SMA  1.06 | DDM  0.84 | SMA  0.428 | DDM  0.365 |

**Supplementary Table 2: Yields of CD81 obtained with different expression and solubilisation conditions.** The yield of yeast cell pellet obtained when inducing CD81 expression at different OD_600_ values. The yield of membrane preparations extracted from these cells following cell lysis using an Emulsiflex-C3 cell disrupter (mean ± sem, n=2). Yield of purified protein following solubilisation using either 2.5% (w/v) SMA 2000 or 2% (w/v) DDM, followed by Ni-NTA affinity chromatography and concentration using a centrifugal concentrator (n=1).

**Supplementary Figure 1**


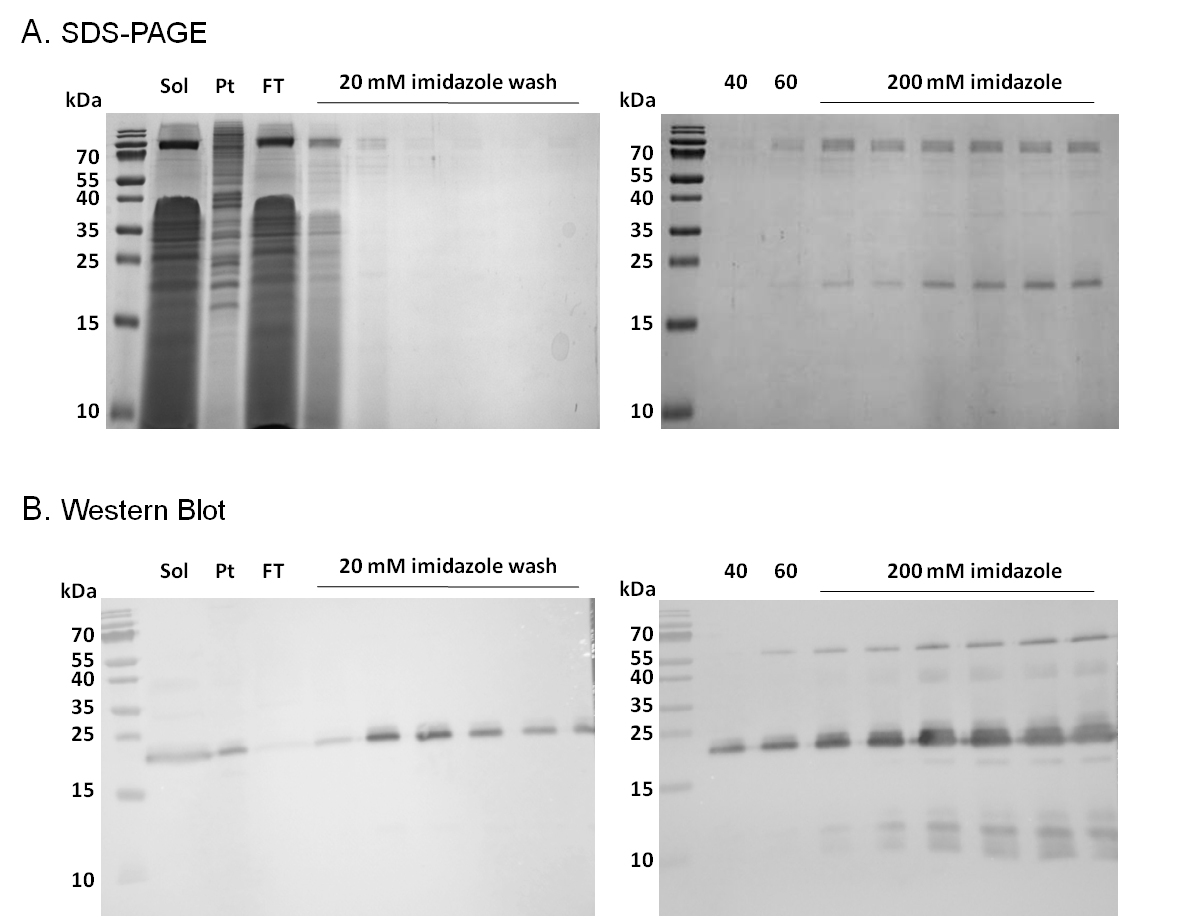


**Supplementary Figure 1.** **Initial purification of SMALP-encapsulated CD81.** Solubilised CD81 was mixed with Ni-NTA resin (100µl bed volume (bv) per ml solubilised protein) overnight at 4°C, then transferred to a gravity flow column and the flow-through collected. The resin was washed several times with 10 bv buffer containing 20 mM imidazole, followed by 10 bv containing 40 mM imidazole and 1 bv containing 60 mM imidazole. Protein was eluted in ½ bv fractions supplemented with 200 mM imidazole. Sol is total solubilised protein, Pt is insoluble protein, FT is flow-through. A) SDS-PAGE analysis of each step of purification, stained with Instant Blue. B) Western blot analysis of the same purification fractions using an anti-CD81 primary antibody.

**Supplementary Figure 2**


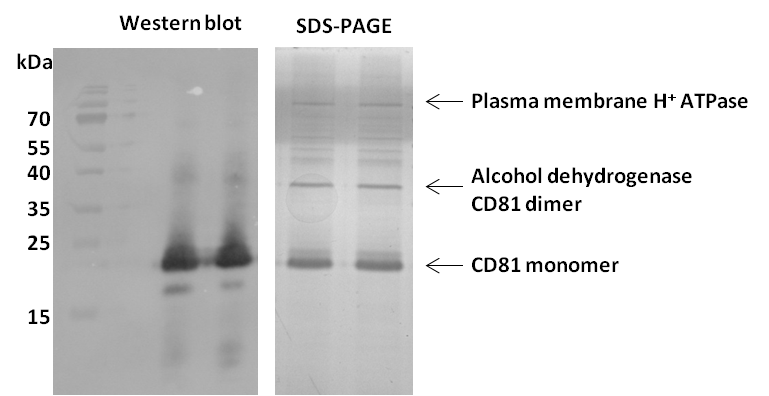


**Supplementary Figure 2.** **Contaminants co-purified with CD81.** Western blot and SDS-PAGE of CD81-SMALP following Ni-NTA affinity chromatography. Several contaminants co-purify with CD81. Those labelled with arrows were identified by mass spectrometry analysis of the bands extracted from SDS-PAGE.

**Supplementary Figure 3**

**Supplementary Figure 3.** **Purification of DDM-solubilised CD81.** A) CD81 solubilised with 1% (w/v) DDM and 0.1% (W/v) CHS was incubated with Ni-NTA resin overnight at 4°C, then transferred to a gravity flow column and the flow-through collected. The resin was washed several times with buffer then eluted with buffer supplemented with 300mM imidazole. Memb is total membrane, Sol is total solubilised protein, FT is flow-through. Samples were run on SDS-PAGE and stained with Instant Blue. B) Purified CD81-DDM samples were assayed by ELISA for binding to conformation sensitive anti-CD81 antibodies or HCV glycoprotein E2 binding.

**Supplementary Figure 4**


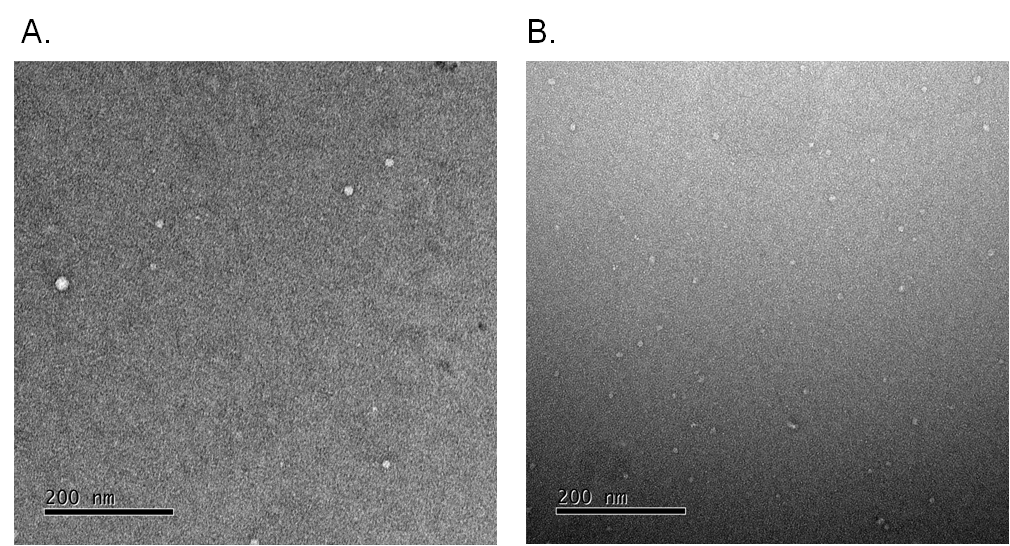


**Supplementary Figure 4.** **Negative stain electron microscopy of CD81-SMALP following SEC.** Representative images obtained when 10 µl of CD81-SMALP from (A) peak 1 or (B) peak 2 following size exclusion chromatography, was immobilised on negatively charged copper grids and stained with 2% uranyl acetate. Grid was imaged on the JEOL2100+ TEM at 60,000 X magnification (at Warwick University Imaging Facility).
